# Why we should talk about option generation in decision-making research

**DOI:** 10.3389/fpsyg.2013.00555

**Published:** 2013-08-23

**Authors:** Annemarie Kalis, Stefan Kaiser, Andreas Mojzisch

**Affiliations:** ^1^Department of Philosophy, Utrecht UniversityUtrecht, Netherlands; ^2^Department of Psychiatry, Psychotherapy and Psychosomatics, Zurich University Hospital of PsychiatryZurich, Switzerland; ^3^Institute of Psychology, University of HildesheimHildesheim, Germany

**Keywords:** decision-making, option generation, option, goal, plan, action representation

## Abstract

Most empirical studies on decision-making start from a set of given options for action. However, in everyday life there is usually no one asking you to choose between A, B, and C. Recently, the question how people come up with options has been receiving growing attention. However, so far there has been neither a systematic attempt to define the construct of “option” nor an attempt to show why decision-making research really needs this construct. This paper aims to fill that void by developing definitions of “option” and “option generation” that can be used as a basis for decision-making research in a wide variety of decision-making settings, while clarifying how these notions relate to familiar psychological constructs. We conclude our analysis by arguing that there are indeed reasons to believe that option generation is an important and distinct aspect of human decision-making.

## INTRODUCTION

The current literature on human decision-making is largely focused on the question how people implement their goals by deciding between different possibilities for action and translating these decisions into action. Traditionally, empirical studies on decision-making start from a set of options provided by the experimenter. Participants are, for example, provided with a range of buttons they can press; or they are asked to choose one from a set of different objects, rewards, or possible solutions to a problem. However, in everyday life there is usually no one asking you to choose between A, B, and C (Woll, 2002; Smaldino and Richerson, 2012). For example, when you miss your train you need to decide how you will spend the time until the next train arrives – but the different options are not presented to you as buttons to press. Rather, you have to generate these options yourself.

The focus in previous empirical research on externally provided options means that there are questions concerning decision-making that are frequently overlooked: how do people determine what they can possibly do at any given moment? How do they come up with “options” for action? Recently, these questions have given rise to some theoretical papers (Kalis et al., 2008; Smaldino and Richerson, 2012) and a small, but growing body of empirical work on option generation (Keller and Ho, 1988; Klein et al., 1995; Raab et al., 2009; Ward et al., 2011). However, so far there has been no systematic attempt to define the notions of “option” or “option generation.” This is problematic firstly because as we will show, different authors implicitly use the terms in different ways, making their results hard to compare. Although we do not claim that everyone studying option-generation mechanisms should use the same definition, one of the aims of the current paper is to argue that there are several aspects that any plausible definition of real-world option generation should cover. Thereby, it should be clarified in advance that the definitions we propose are not themselves based on empirical findings (of which there are few), but that they are grounded in a conceptual analysis of decision-making and should be seen as normative guidelines for structuring future empirical studies on option generation.

Secondly, unclarity about what these terms mean raises the question whether these concepts are really needed – is it not the case that everything interesting about option generation is already covered by familiar constructs such as goals and plans? Therefore, our second aim is to analyze to what extent decision-making research really needs constructs such as “options” and “option generation.”

The theoretical analysis developed in this paper aims to contribute to existing stage models of decision-making (Heckhausen and Gollwitzer, 1987; Gollwitzer, 1990; Ernst and Paulus, 2005; Heckhausen and Heckhausen, 2008; Kalis et al., 2008). These models distinguish different stages or aspects of the decision-making process. Stage models do not presuppose that decision-making is a linear process; some argue instead that it consists of iterative cycles, each cycle consisting of different phases (Maani and Maharaj, 2004). In earlier work (Kalis et al., 2008), we briefly noted that stage models might need to be expanded with an option-generation stage; in this paper, we develop this proposal in full detail. In the first sections of the paper, it will be argued that although there is increasing attention in decision-making research for the stages preceding evaluation and choice, different authors use the terms “option” and “option generation” in different ways. Also, it will be shown that existing research on option generation restricts itself to certain specific settings, thereby overlooking major areas of decision-making. We will then propose definitions of the terms “option” and “option generation” that can be used as a basis for research in a wider variety of decision-making settings, while clarifying how these notions relate to familiar psychological constructs. This analysis will make clear why option generation should be seen as an important and distinct aspect of human decision-making in real-life settings. The final part of the paper provides suggestions on how to move forward in research on option generation.

## OPTION GENERATION: AN OVERVIEW

The literature on decision-making uses the terms “option” in at least four different ways, differing in level of abstractness. A very abstract notion of options can be found in, for example, Ward et al. (2011), who define options as statements that are relevant to the attainment of a certain goal. This means that in their view, options can be predictions, desires, perceived possibilities, or plans, to name just a few possibilities. In their empirical study, Ward et al. (2011) examined the options generated by police officers in a simulated situation of potential threat. A statement like “I think the suspect has a gun” is in their study counted as an option because it is a statement that is relevant to the attainment of a certain goal (namely preventing the suspect from using lethal violence).

A second, more restricted definition of options is found in work on hierarchical reinforcement learning. Here, the term “options” refers to “abstract actions”: temporally extended sequences of lower-level actions (Sutton et al., 1999; Botvinick et al., 2009). This means that lower-level actions such as cutting an onion, getting butter from the fridge, frying the onions, and so on can be grouped into an option such as “making dinner.” This proposal aims to do better justice to the hierarchical structure of action descriptions and provides a framework for studying the role of reinforcement learning in more complex forms of behavior.

Thirdly, problem-solving studies use the term “option” in an even more restricted sense, referring to “steps that could be taken in solving a problem” (Keller and Ho, 1988; Klein et al., 1995). The range of options is then determined by the range of actions that are allowed within the context of the problem. For example, in their study on option generation in chess, Klein et al. (1995) asked participants to generate possible moves in response to a certain situation on the chess board. In this study, options are conceptualized as possible chess moves as judged by the participants. Similarly, in solving a problem like the Tower of London (Shallice, 1982), options would be the different moves allowed within the puzzle.

The most fine-grained notion of “options” can be found in the work of Raab and colleagues (Johnson and Raab, 2003; Raab and Johnson, 2007; Raab et al., 2009) on option generation in sports situations. In their work, they investigate specific ways in which a movement in a sports situation can be completed. In a study on basketball, for example, the options measured are different ways to throw a basketball with regard to “velocity, release angle and spin” (Raab et al., 2009, p. 52). Their work aims to show that option generation does not require elaborate cognitive processing and can be understood by combining the theories of direct perception (Michaels and Carello, 1981) and bounded rationality (Gigerenzer and Selten, 2002).

This brief overview shows that the different areas of research on option generation differ widely in focus; as said this makes the results of different studies on option generation hard to compare. One could ask whether a study that examines “statements relevant to goal attainment” is really about the same thing as a study that examines different ways to throw a basketball. Precisely because the field of option generation is in an early stage, it seems important to aim for clarity regarding the question what option generation is, and what connects the different studies that examine it.

While one problem is that existing studies vary widely in their use of the terms, another problem is that the areas of decision-making covered in most existing studies on option generation are limited. Almost all studies focus on decision-making situations that have stringent constraints (solving a chess problem, judging a potentially violent situation as a police officer, throwing a basketball). The most important reason for this seems to be a methodological one: constraining the range of options participants work with increases control over the experimental situation. However, this also implies that the experimental settings in which decision-making is studied are far removed from everyday decision-making situations, where the range of options is often wider and less strictly constrained than the range of possible moves in a chess problem or in sports. In order to be able to cover also such underconstrained situations in empirical research, it is first needed to see more clearly how options for action in real life could be understood. Note that the proposal developed below does not have the aim to replace current uses of the term described above, but to provide a more systematic conceptual analysis of options and option generation that can be used in broad areas of decision-making.

## DEFINING OPTIONS

Before discussing option generation, we first want to focus on the simple notion of an option. As said, we believe that there are two aspects that any plausible definition should address. The first is that (and this is in line with what has been implicitly assumed in most empirical studies) an option for action should be understood as a *mental representation* of something the agent could do: as a representation of an action. In this respect, it differs from the closely related notion of an affordance (Gibson, 1977; Norman, 1999) used in the fields of ecological psychology and human–machine interaction. According to most authors, an affordance is not a mental state, but an action possibility situated in between a specific person and his/her environment. “Options” also have a broader scope than affordances, as we might generate highly abstract or future options that are not present in the environment. Acknowledging that an option is a *mental state* implies, for example, that if one refers to options as “possible solutions to a problem,” one should keep in mind that these are always possible solutions seen from the point of view of the decision-maker. Of course, options can vary widely in quality and feasibility; the point made here is that something can be an option even if it is not an objective possibility. Let us say that you generate the option of going to see the Nachtwacht at the Rijksmuseum tomorrow, not knowing that it will be closed. The fact that seeing the Nachtwacht tomorrow is impossible does not mean that you did not generate an option: it just means that you generated an option with zero feasibility.

Secondly, an option is not just any representation of an action, but an action representation one might decide on, and that one considers deciding on; it is a choice alternative. This means that options should be distinguished from action representations that are involved in, for instance, idly imagining a certain action taking place. It also implies that contrary to what is sometimes stated in the literature (Sprenger and Dougherty, 2012), option generation is different from hypothesis generation, where one generates “possible explanations” for a certain event (Gettys and Fisher, 1979). Although hypothesizing about, for example, the causes of a problem might certainly affect the options generated for solving the problem, generating hypotheses has nothing to do with action. Of course, the requirement that one sees an action representation as a choice alternative does not need to involve anything like an explicit thought “I believe that I could decide to take a cruise.” Also, taking something into consideration does not need to happen consciously. The only requirement is that in order to be an “option,” an action representation should play the implicit or explicit psychological role of a candidate for action.

We thus, propose to define options as representations of candidates for goal-directed action. In the remainder of this section, we would like to discuss two characteristics of options that play an important role in existing work on option generation: the fact that they are frequently organized in a hierarchical structure and the fact that they have an “evaluative load.” As described in Section “Option Generation: An Overview,” different uses of the term “option” denote different units of analysis (sets of primitive actions, primitive actions, or ways to perform primitive actions). Building on existing stage models of decision-making combined with the idea that action representations are structured in a hierarchical way (Sutton et al., 1999; Manoel et al., 2002; Grafton and Hamilton, 2007; Botvinick et al., 2009), it is plausible to think that decision-making also has a hierarchical, tree-like structure (Botvinick et al., 2009). In many unconstrained decision-making situations, one might first decide on a general course of action on the basis of various abstract options, after which the decision-making process moves down to a more concrete level, involving the consideration of more concrete options. For example, when thinking about where to go on holiday, you might see various options (going camping, going on a cruise, renting a cottage). When that is decided upon, the next question (for instance, where to rent a cottage?) might lead to the generation of more concrete options for action (renting a cottage in France, in Germany, and so on). At the most fine-grained level of analysis, options might be different ways in which a certain movement may be performed, as examined in the studies by Raab et al. (2009). Options, therefore, should be seen as elements of a hierarchical structure of action representations, ranging from the very abstract to the very specific, and ranging from options that can be performed here and now to options that extend over time.

Finally, in most decision-making situations the options generated are not *neutral* representations of an action, but representations that already carry a certain “evaluative load.” After all, options play a role in decision-making: what is at stake is the question what one prefers to do. Generating options is not the same as trying to list all possible things one could do (standing on your head, buying seven cans of tomatoes, buying eight cans of tomatoes...). This does not mean that evaluation must be finalized during the phase of option generation: in most stage models, the evaluation of options is considered to be a separate phase, culminating in the selection of an option (Heckhausen and Gollwitzer, 1987; Ernst and Paulus, 2005). However, work in ecological psychology (Gibson, 1977) suggests that some kind of evaluation might already be taking place while options they are being generated. Such “immediate evaluation” might be the result of processes such as reward learning or somatic marker associations (Damasio, 1999). More specifically, we propose that two distinct values of an option can be distinguished. The intrinsic value of an option refers to the hedonic feeling associated with pursuing the option. In contrast, the extrinsic value of an option refers to its instrumentality for achieving a particular goal (Vroom, 1964; Deci and Ryan, 1985; Higgins and Trope, 1990). For example, for many people practicing yoga has both a high intrinsic and a high extrinsic value because it is a relaxing activity which, at the same time, serves to increase flexibility and health. In contrast, for most people, running 10 kilometers in a cold rain has a low intrinsic value, but may have a high extrinsic value for someone who is trying to stay fit.

## OPTIONS, GOALS, AND PLANS

Our proposal is thus to define options as representations of candidates for goal-directed action. This raises the question: how does the construct relate to more traditional constructs from the decision-making literature like goals and plans, and can it really be distinguished from these existing constructs? In the previous section, we already argued that options should be distinguished from possibilities. We consider the range of possibilities to be the range of action sequences that are physically possible for an agent in the actual world. This notion of “possibility” is very similar to the notion of an affordance (see above, and Gibson, 1977; Michaels, 2003). Options, on the other hand, are representations of perceived possibilities for action. The term “perceived possibility” implies that a decision-maker might be mistaken about what is physically possible – but a representation of such an (incorrectly) perceived possibility is nevertheless an option. In the rest of this section, we will focus on goals and plans, and argue that what distinguishes options from these concepts is their formal role in the decision-making process: the formal role of an option is its being a possible action that is taken into consideration (but not yet decided upon).

### GOALS

An influential definition by Austin and Vancouver (1996) states that goals are:

“internal representations of desired states, where states are broadly construed as outcomes, events, or processes. Internally represented desired states range from biological set points for internal processes (e.g., body temperature) to complex cognitive depictions of desired outcomes… that span from the moment to a life span and from the neurological to the interpersonal” (Austin and Vancouver, 1996, p338).

On this view, a goal is understood as a kind of representation, leaving room for a wide range of states differing in complexity and abstractness. A more recent definition states that goals are “subjectively desirable states of affairs that the individual intends to attain through action” (Kruglanski and Kopetz, 2009, p. 29). Both definitions at first sight seem to lie very close to our proposed definition of an option for action. The important thing to see here is that whereas certain representational *content* (such as the representational content “buy ice cream”) might figure in both goals and options, options are different kinds of representations than goals: both play different roles in the decision-making process.

There are two important differences. First, the second definition mentioned above refers to the fact that goals are states that one intends to bring about. Of course this leaves room for the fact that we might assign different values to different goals, and that some of our goals might clash, leading to ambivalence; what is important here is that in order for a possible state to be one of our goals, we must at least have some *prima facie* intention to realize it. On the other hand, with options this is not the case. Although options do have some *prima facie* evaluative load, it is not the case that an agent intends to realize every possibility that he or she sees. When my goal is losing weight, I might come up with different options for realizing that goal. I might start exercising daily, I might go on a diet, I might apply for cosmetic surgery. The fact that I generate those action representations as options does not mean that I want to bring them about: I probably find some options more attractive than others; what I want to do remains to be decided upon. The second difference is that options are by definition representations of *actions*, whereas goals are not. For example, one could adopt a goal such as “being happy”: this goal is not the representation of an action, but of a certain state of mind. Another example is someone having the goal “the neighbor saying sorry for his rude behavior.” This goal is not a representation of an action either, but a representation of a certain external outcome.

### PLANS

A second question is whether options are in any important sense different from action plans. The literature on planning largely focuses on planning as required for effective goal achievement. Despite the numerous definitions of “plans” and “planning” in the psychological literature, there is considerable convergence. Plans can be defined as mental representations of behavioral sequences for reaching a goal (Goel, 2002) or as cognitive structures that allow an agent to transform his decision into action (Gollwitzer, 1990). In the same vein, the philosopher Michael Bratman developed an influential theory of planning, defining plans as coherent structures of commitments that are directed toward the attainment of a goal (Bratman et al., 1988; Bratman, 1989, 1999). Plans as structures of commitments can be of varying complexity: a simple plan would be “take a different route to work today,” whereas a complex plan would be “open a record store.” It is clear that plans are thus related to goals: plans are strategies for reaching a goal, and the structure of a plan is derived from the way in which the different steps contribute to the fulfillment of the goal.

But do options differ from plans? After all, both options and plans might be seen as strategies (i.e., means) for attaining a goal. However, the literature on planning suggests that there are some aspects in which plans are different from options. Firstly, a plan is often considered to be something characterized by some form of commitment (Bratman, 1999). In most stage models of decision-making, plans come into play *after* one has selected a certain option for action (Gollwitzer, 1990): a plan is an answer to the question how to transform the selected option into action. Exceptions are cases of planning that are not directly linked to a decision, such as planning the day ahead. But even in such cases, planning seems to be accompanied by an intention to actually execute the plan (on the relation between plans and intentions, see Pollack, 1990; Bratman, 1999). The only cases where plans are made without a corresponding intention are cases of hypothetical planning such as: “if I were to rob a bank I would do it like this…” – but even here one could argue that there is a “hypothetical intention” involved. On the other hand, as said above in the discussion about goals, seeing something as an option for action does not imply any form of commitment or intention. Secondly, options are singular action descriptions (although these can vary in complexity and be embedded in a hierarchical structure), whereas plans consist of sequences of different steps that “guide” a person toward the attainment of a goal (first I will do this, and then I will do this, and then…). To conclude, while there is thus overlap in the application of the terms “options” and “plans,” the notion of an option refers to a perceived possibility that is taken into consideration, whereas the notion of a plan generally refers to a sequential structure of action steps characterized by a certain commitment.

In sum, the main point so far has been that options are not identified by a specific representational content: as we have seen a representation with the content “start exercising daily” could be either a goal, a plan, or an option. What distinguishes options from goals and plans is their formal role in the decision-making process: options are possible actions that are taken into consideration (but not yet decided upon). Now that we know how to define the notion of an option, the next question is: what could it mean to *generate* options for action?

## DEFINING OPTION GENERATION

The term “option generation” is mostly used very generally, to refer to the idea that decision-makers come up with options as a result of certain psychological processes. However, Raab et al. (2009), for example, seem to distinguish option generation from option perception, restricting the latter notion for those situations in which one directly “sees” options in the environment. Notwithstanding the importance of this research, at least on our definition of options, the notion of option perception is not really appropriate: when one defines options as mental representations, this implies they are not merely part of the environment: even if the environment suggests (affords) a possibility, in order for that possibility to become an option, some mental activity is needed. Therefore, perception might be a misleading term. Our proposal is to define option generation as the formation of mental representations of candidates for goal-directed action. This basic definition does not yet answer the question whether option generation should be seen as a distinct psychological process, or whether processes such as memory retrieval, problem solving, and so on provide us with options, for which the term “option generation” is a shorthand description. This unclarity is problematic because it makes it hard to see whether we really need a new construct of option generation at all. In this section, we will develop a proposal on how to analyze the concept of option generation; in the next section, it will be argued that whereas option generation is probably not a separate psychological process, it nevertheless needs to be studied in its own right.

The first problem is how to demarcate the “domain” of option generation: is every action preceded by an option-generation process? Or does option generation only take place when we are consciously deliberating about what to do? It seems plausible to say that there is option generation going on if and only if there is decision-making about action going on. Given that options were defined as representations of candidates for goal-directed action, we are not concerned here with people generating possibilities that are not related to action (such as generating possible events that might happen in the future). Secondly, the demarcation criterion implies that there is no option generation at work in behavior that is completely automated, habitual, and stimulus-driven: for example, if one sees a word in front of one’s eyes, one will automatically read it (Stroop, 1935). In such situations, no decision-making is involved^1^, and, therefore, no option generation takes place either. However, just as there are many forms of implicit decision-making, this also applies to option generation. Option generation does not need to take the form of conscious, explicit “thinking about possibilities”; there are probably many types of real-life decision-making where options just “pop into your head” or are directly suggested by your environment. However, one might ask whether the kind of processes studied in the work by Raab et al. (2009) would really count as the generation of options on our proposed definition. As said, Raab et al. (2009) investigated decision-making in sports situations, for example, how people “generate and perceive options for controlling rotation” (p. 57) in performing a double somersault. Although the authors claim that such behavior involves decision-making because it is goal-directed behavior, the decision-making (and option generation) at stake here is certainly of a highly implicit kind.

This leads to the question how different kinds of option-generation situations could be distinguished. It seems fruitful to place option-generation situations along a continuum that on the one end has situations where decision-makers have an explicit goal they are trying to attain, and, on the other hand, situations that are completely open and not guided by any explicit goal. In a situation representing the first extreme, option generation amounts to determining different possible means to attain an explicit goal. This is thus the kind of top-down option generation usually studied in work on problem-solving: option generation in the context of, for example, a chess problem (Klein et al., 1995), a parking problem (Adelman et al., 1995), or as a response to one’s New Year’s resolution to improve one’s work-life balance. In such situations, options are means to a certain end, and the question how options are generated is also the question how means are adopted (Kruglanski and Kopetz, 2009). On the other end of the continuum are situations where there is no explicit goal: here the process of option generation takes place in a bottom-up fashion and is much less constrained. One possible example is the situation where you unexpectedly have a free day in front of you (let us say you had obligations that were canceled at the last moment) and you ask yourself: what shall I do with my time? This type of option generation has so far hardly been studied at all, as it is particularly difficult to examine in empirical research due to it being heavily underconstrained: the agent not only needs to determine what he could do, but also what he would like to achieve. Nevertheless, this kind of option generation plays quite an important role in our everyday life.

One might object that what happens in these latter cases is that the agent first sets a goal. However, this seems an overly linear approach to the decision-making process. It does not seem plausible to say that an agent *always* first needs to set a goal before generating options. As Kruglanski and Kopetz, 2009, p. 35) argue: “a means may activate the corresponding goal.” Let us say that while reflecting on your free day your eye falls on the movie listings in the newspaper: this might activate your goal of seeing that new movie with Angelina Jolie, and result in your generating the option of going to the cinema. Option generation in such a situation consists in perceiving a certain affordance or opportunity for action (namely, going to the cinema) in your environment. This is likely the kind of example Raab et al. (2009) may have had in mind when speaking of “option perception.” However, strictly speaking, it is not the option that is perceived, but the opportunity or affordance; the option itself is the mental state resulting from such perceptual processes. It is important to note that even though the agent in such situations does not have an explicit goal, research so far suggests that option generation is probably strongly guided by the agent’s implicit goals, preferences, or motives (Hassin et al., 2008; Custers and Aarts, 2010). After all, when you would have a dislike for movies, seeing the movie listings in the newspaper would not lead you to generate the option of going to the cinema. Or to give another example, individuals who have a high-affiliation motive will be likely to generate affiliation-related options, such as calling a good friend, whenever they have spare time. In contrast, individuals who have a high-achievement motive will be more likely to generate achievement-related options, such as working on a manuscript. In these examples, there is no explicit goal, but option generation is guided by implicit (i.e., more or less unconscious) goals or motives (Schultheiss and Brunstein, 2010).

## OPTION GENERATION, CREATIVE COGNITION, AND PLANNING

As said, option generation is usually taken to refer to every process that provides a decision-maker with options. So far none of the studies on option generation claim that option generation should be seen as a distinctly identifiable psychological process that is completely independent of familiar processes such as memory retrieval, perception, or problem-solving. Research so far suggests that memory retrieval, in particular, plays a substantial role in option generation (Klein et al., 1995; Johnson and Raab, 2003; Kaiser et al., 2013). As noted by Kalis et al. (2008), it is likely that the importance of each process depends on the familiarity and complexity of the situation. Thus, it is plausible that in unfamiliar or complex situations, option generation is more effortful, and, therefore, relies more on processes associated with executive function, whereas in familiar or well-constrained situations, option generation requires less effort and might rely more on processes associated with (more or less) automatic retrieval from long-term memory. However, there is still reason to think that option generation cannot be understood just by looking at what is known about those familiar processes. This is because in generating options these processes play a certain specific role – namely the role of providing agents with representations of possible choice alternatives. This “role” can be clearly distinguished from the role these processes have in other psychological contexts. For example, when you are at the station with an hour to spare, memory retrieval could very well play a part in how you generate options (visiting that coffee bar, buying a newspaper over there, etc.). But how exactly and under what conditions memory plays such a role in everyday option generation might not be found out just by studying memory retrieval in general. However, one might object: could not option generation just be described as a form of creative cognition or of planning? We will discuss these processes and their relation to option generation in the next section.

### CREATIVE COGNITION

Creative cognition is generally accepted as having two defining aspects: it is the generation of both novel and adequate products of thinking (Sternberg, 1999; Dietrich, 2004; Ward, 2007). Looking at this definition, it becomes clear that option generation differs from creative cognition in two important ways: creative cognition is always about generating *novel* products whereas option generation is not, and option generation is always about action, whereas creative cognition is not. Regarding the first point, it is of course true that generating novel solutions or ideas might greatly contribute to option generation. But there is no reason to think that option generation requires creative thinking, nor that creative thinking always leads to optimal option generation. Regarding the second point: creative cognition might result in thoughts or ideas that have nothing to do with actions. For example, the classic remote associates test (Mednick and Mednick, 1967) measures creative cognition by asking subjects to generate associations in response to a set of three words (such as, for example, the word “plumber,” “tobacco,” and “tube,” which together suggest the word “pipe”). The test measures subjects’ responses in terms of creative insight and adequacy. This kind of cognitive task is completely different from the task of generating options for action. There are also creativity tasks that are more closely related to action: an example is the Brick Test (Guilford et al., 1978), which requires participants to list as many as possible uses for a brick. However, even in this task what is measured is not how relevant the uses generated are for a person’s goals, but how innovative they are. All this naturally still allows for the fact that option generation might very well lead to creative products of thinking: however, the formal role of option generation clearly differs from the role of creative cognition.

### PLANNING

In the same vein, option generation differs from planning. In most cases of planning, agents are implementing a certain decision (for exceptions, see Plans): the question at stake in planning is how to realize one’s goals in action. This means that in contrast to option generation, planning is characterized by commitment to a choice of action (Bratman, 1999). However, given what was said earlier about iterative cycles of decision-making and the hierarchical structure of action representation, planning might in turn “require” option generation. This can be illustrated with an earlier example: having decided to rent a cottage (and thus, planning to rent a cottage), you might, in turn, need to generate options with respect to the question *where* to rent a cottage. However, in such cases, the agent is not committed to the options generated (a cottage in France, in Spain, etc.) even though he is committed to the general plan “renting a cottage.” One could say that the “type” of option generation probably shifts as the decision-making process proceeds: in the early stages of deciding about something like a holiday almost everything is open: in this stage, option generation will probably be relatively unconstrained and bottom-up. In later stages, when the decision-making has progressed to a more concrete level (it has been decided what type of holiday, and what type of destination), option generation becomes more constrained and top-down, guided by the goals that have been set in earlier stages.

## CONCLUSION

According to our proposal, research on option generation could find a common conceptual starting point in the idea that options are mental representations of candidates for goal-directed action. Option-generation processes are proposed to be those cognitive processes fulfilling a specific role in the decision-making process, namely providing the agent with such representations. In this final section, we hope to show how these definitions could provide a sound basis for empirical studies on everyday option generation.

Firstly, by defining options as *mental representations* and not conceivable possibilities, the question comes to the fore how we come from all conceivable possibilities to things seen as options. At any point in time, there is an almost infinite number of things we could do. Nevertheless, we do not consider most of them as options for action (Galotti, 2007; Smith, 2010; Smaldino and Richerson, 2012). What kind of constraints are applied here? And why do some people apply stricter constraints than others? One might hypothesize that differences in the personality trait “openness to experience” (McCrae and John, 1992; McCrae, 1996) would correspond to differences in constraints applied in option generation: some people just see more options than others.

Secondly, our proposal has been to demarcate the domain of option generation by seeing it as an aspect of decision-making, thus excluding fully habituated and automated types of cognition. This of course still leaves open the question: when do we speak of decision-making? This is a question that cannot be solved within the scope of the present paper – in fact, it might not have a clear-cut answer at all. However, given our aim to develop a theoretical basis for research on everyday decision-making, we believe that the focus of option-generation research should be on situations that are clear cases of decision-making: thus, on situations where we can ask ourselves: what could I do?

If we start from such a conceptual basis, how should research on option generation proceed? As is being acknowledged by those working in this field (Galotti, 2007; Raab et al., 2009), the next hurdle to take is to develop option-generation studies encompassing a higher level of real-world complexity: thus, studying everyday decision-making situations that are heavily underconstrained. As a first step, we have recently developed an experimental paradigm in which participants are asked to generate options for simple real-world scenarios^2^, and to subsequently decide among the generated options (Kaiser et al., 2013). Our results show that decisions between self-generated options can be clearly distinguished on both the behavioral and the neural level from decisions between externally provided options. These findings provide empirical support for our claim that we need to be cautious when transferring the results of previous studies in which participants had to decide between options provided by the experimenter to real-life situations where individuals decide between self-generated options. Furthermore, in order to approach real-world complexity, it is important to develop paradigms that work with the decision-maker’s own goals. So far, most empirical paradigms on the processes mentioned above work with goals that are provided by the experimenter.

In addition, the question how different psychological processes fulfill the task of providing an agent with options for action, and which processes are involved in which kinds of option generation, are largely unstudied. One of the major questions, therefore, is how these different processes interact in the generation of options for action. What determines whether options are generated from memory, automatic perceptual processes, or by creative cognition? How do situations where a goal is given, differ in this respect from situations where the agent does not have a clear goal? What does option generation look like in cases of conscious (effortful) decision-making, compared to more automatic (effortless) forms of decision-making?

More generally, option generation sets the constraints for the decision-making process as a whole: if you do not see that a certain actual possibility is an option, you will never be able to act on this possibility. This means that the options we generate (or do not generate) partially determine the *quality* of the decision that will be made (Sprenger and Dougherty, 2012). This is important because in generating options for action, decision-makers do not attempt to generate as much or as crazy options as possible: they try to generate those options that enable them to make good decisions. Therefore, an important question is how different factors affect option quality. This is particularly relevant for the study of dysfunctional decision-making: is it, for example, possible to identify dysfunctional processes of option generation in terms of either the quantity (too many, or too few) or the quality of options generated (cf. Kalis et al., 2008)? And if so, how do such dysfunctional processes play a role in different psychopathological symptoms related to decision-making?

Finally, another promising avenue for research is to study the role of option generation in the everyday phenomenology of agency (Farrer and Frith, 2002; De Vignemont and Fourneret, 2004; David et al., 2008). For example, it is possible that our sense of agency might be stronger if one is acting on the basis of self-generated options than if one is choosing between externally provided options. After all, what seems characteristic about our experience of making decisions is that we do not merely choose between different things that are presented to us: we can at least some of the time *actively* come up with ideas about things we could do. So far, attention has been focused on the role of choosing or deciding as being important for a sense of free agency: “I feel free because I can choose between different things.” Research on option generation, however, might indicate that option generation provides another important aspect of the experience of free agency: “I feel free because I can see different possibilities.” Clearly, future research is called for to better understand the processes underlying option generation and the intricate relationship between options, goals, and plans. We hope that our paper serves to stimulate further research in this largely uncharted territory of human decision-making.

## Conflict of Interest Statement

The authors declare that the research was conducted in the absence of any commercial or financial relationships that could be construed as a potential conflict of interest.

## Footnotes

1 However, a reader fluent in both Hebrew and English might still have to decide between reading the word from left to right or from right to left. This shows that even in many cases of apparently automatic behavior, some decision-making process might be taking place (we are grateful to one of the reviewers for pointing this out).

2 To give an example, one of the scenarios used was: “You have missed your train. You have to wait for one hour until the next train leaves. What could you do?” (Kaiser et al., 2013).
